# Glioma of the optic nerve and chiasm: a case report

**DOI:** 10.5935/0004-2749.2021-0246

**Published:** 2022-07-04

**Authors:** Camila Batista Pas, Guilherme Henrique Tanajura, Jair Giampani Junior, Amanda Garcia de Brito

**Affiliations:** 1. Departamento de Oftalmologia, Hospital Universitário Júlio Müller, Cuiabá, MT, Brazil

**Keywords:** Glioma, Optic nerve neoplasms, Optic chiasm, Astrocytoma, Magnetic resonance imaging, Visual Acuity, Case reports, Humans, Glioma, Neoplasias do nervo óptico, Quiasma óptico, Astrocitoma, Imageamento por ressonância magnética, Acuidade Visual, Relatos de casos, Humanos

## Abstract

This article reports the case of an 11-year-old male patient with a history of proptosis and low progressive visual acuity in the left eye. He presented with a best corrected visual acuity of 20/25 in the right eye and light perception in the left eye. Exotropia and limitation in adduction were observed in the left eye. On automated perimetry, inferiortemporal quadrantopsia was observed in the right eye, while total scotoma was observed in the left eye. On magnetic resonance imaging, there was an expansive lesion in the left optic nerve, extending to the brainstem with chiasmatic involvement. This article aims to report a case of optic pathway glioma, as well as to discuss its clinical findings and their interconnection with the current literature.

## Abstract (pt)

Este artigo relata o caso de um paciente do sexo masculino, 11 anos de idade, com história de proptose e baixa de acuidade visual progressiva. Ao exame oftalmológico apresentava melhor acuidade visual de 20/25 em olho direito e percepção de luz em olho esquerdo. Existia exotropia e limitação à adução no olho esquerdo. À campimetria automatizada, observou-se quadrantopsia temporal inferior em olho direito e escotoma total em olho esquerdo. À ressonância magnética, evidenciou-se lesão expansiva em trajeto do nervo óptico esquerdo estendendo-se até região do tronco encefálico, com acometimento quiasmático. O objetivo deste artigo é relatar o glioma de vias ópticas, bem como discutir os achados e sua interligação com a literatura atual.

## INTRODUCTION

Optic pathway gliomas (OPGs) represent about 2%-5% of pediatric brain tumors and occur in young children during the first and second decades of life^(1,2)^. These lesions can grow anywhere along the optic pathway, from the optic nerves to the occipital cortex, including the optic-chiasmatic and hypothalamic regions^(3)^.

The vast majority of these optic pathway tumors are histologically classified as pilocytic astrocytomas and pilomyxoid astrocytoma (grade 1 and 2, respectively, according to the World Health Organization). In pediatric patients with optic-chiasmatic and hypothalamic gliomas (OCHGs), the most common histological type is pilomyxoid astrocytoma^(2,4)^.

The association of optic gliomas with neurofibromatosis type I (NF-1) is important and well-described in the literature, with a prevalence ranging from 15%-21%^(5,6)^. The most common clinical presentation of OPGs, when symptomatic, is a decrease in the visual acuity; other common findings include abnormal pupillary function, dyschromatopsia, optic atrophy, and proptosis^(7)^. The patient may also present with endocrinological disorders and hydrocephalus in the optic-chiasmatic region.

The definitive diagnosis is made via anatomopathological examination, but nuclear magnetic resonance (MRI) has very characteristic findings which are enough to support a diagnosis^(8)^.

The purpose of this article is to report a case of OPG extending to the brainstem, as well as to discuss the clinical findings and their interconnection with the current literature on the topic.

## CASE REPORT

**Figure 1:**
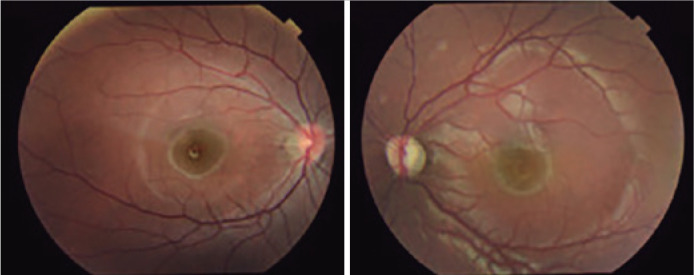
Tilted optic discs with crescents of temporal atrophy and pale staining in the left eye disc.

**Figure 2:**
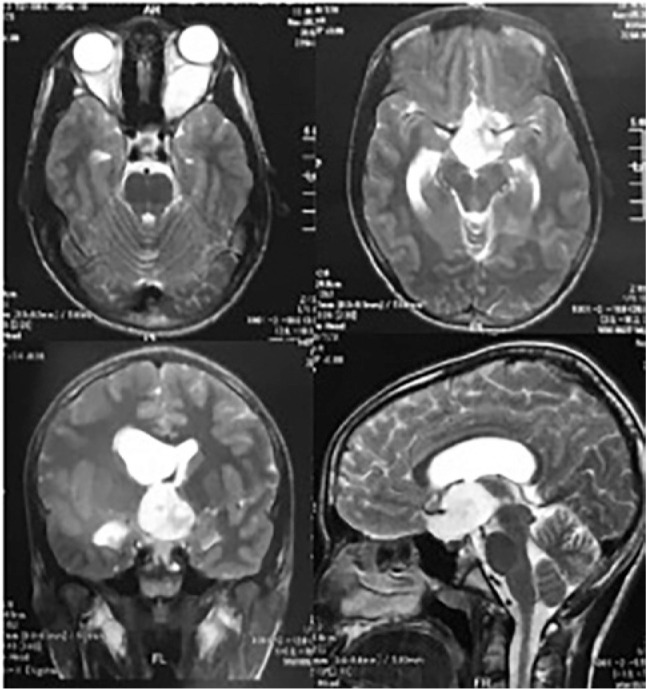
T1-weighted Gd-enhanced magnetic resonance images demonstrating a brightly enhancing optic pathway glioma involving the optic chiasm and hypothalamus with brainstem extension.

The patient is, V.G.C.S, an 11-year-old, previously healthy male who presents with a 2-year history of proptosis and progressive low visual acuity, worse in the left eye. There was no previous history of ophthalmologic disease. Ophthalmological examination showed better visual acuity of 20/25 in the right eye (RE) and light perception in the left eye (LE). Proptosis, limitation of adduction, hypertropia, and relative afferent pupillary defect were found in the LE. Biomicroscopy, tonometry, and gonioscopy findings were essentially normal in both eyes (BE). Fundoscopy of BE showed tilted optical discs with crescents of temporal atrophy and pallor in the LE disc (Figure 1). Optical coherence tomography (OCT) of the RE showed a sectoral nasal decrease in the macular layer of ganglion cells and in the layer of nerve fibers superior to the disc. OCT in the LE showed diffuse reduction in the thickness of both layers. There were no pathological systemic findings. Automated perimetry and MRI of the skull and orbits were requested. During perimetry, inferior temporal quadrantanopsia was observed in the RE and total scotoma was observed in the LE. MRI revealed an expansive and fusiform lesion (7.1 × 2.4 cm across the largest diameter) along the path of the optic nerve to the brainstem region with heterogeneous contrast uptake, suggestive of OPG, as well as asymmetrical lateral ventricles, larger on the right (Figure 2). The patient was referred to a neurosurgical service, with primary surgical management considered. Microsurgery with resection of an orbital and intracranial lesion was performed 1 month after diagnosis, without major complications. Histopathological diagnosis revealed grade 1 pilocytic astrocytoma based on the World Health Organization classification. Two months after surgery, the patient presented with a better visual acuity of 20/20 in the RE and light perception in the LE, orthotropy, without changes in ocular motility, with enophthalmos, and eyelid ptosis in the LE. Visual field testing revealed temporal hemianopsia in the RE and total scotoma in the LE.

## DISCUSSION

The patient had a clinical and ophthalmological condition suggestive of an expansive orbital lesion, marked by severe proptosis and significant visual loss. Automated perimetry suggested intracranial involvement in a chiasmatic region; imaging was the basis for the diagnosis of optic-chiasmatic glioma.

The association between optic gliomas and NF-1 is widely described in the literature, with a prevalence of 15%-21%^(5,6)^, but this was absent in the reported case. In non-NF-1 patients, the optic chiasma and hypothalamus were reported as the most common sites of involvement^(2,3)^, which is consistent with the clinical presentation of this case.

Because of its variable evolution, treatment for this disease is still controversial in the literature, with management options including observation, surgery, chemotherapy, and radiotherapy^(9)^. Chemotherapy is considered the treatment of choice for optic/hypothalamic gliomas, but adjunctive treatment with radiotherapy can also be useful in tumors refractory to chemotherapy^(2)^. Indications for tumor excision usually include severe visual impairment caused by tumor compression in the visual pathway, progressive and severe proptosis, tumor extension to the chiasma and hypothalamus causing endocrine dysfunction, and mass effects on surrounding structures with or without hydrocephalus, along with unfavorable prognostic factors (e.g. sporadic optic gliomas and involvement of the optical tracts/radiation)^(1,2,7)^.

Some authors have linked OCHGs with poor prognosis, with a higher rate of morbidity compared to prechiasmatic optic nerve gliomas. Predictors of poor prognosis include early age of symptom onset (i.e., between 1 and 3 years old), place of origin (i.e., postchiasmatic), presence of diencephalic syndrome, and non-NF-1 patients^(2,3,5,9)^.

The patient had ophthalmological manifestations resulting from nerve and chiasmal compression. Due to severe proptosis, major visual loss, and tumor extension to the optic chiasma, we opted for surgical excision of the tumor. The patient should be monitored continuously to rule out tumor recurrence^(10)^, in addition to multidisciplinary monitoring with pediatric oncologists, endocrinologists, and psychologists.

## References

[1] Binning MJ, Liu JK, Kestle JR, Brockmeyer DL, Walker ML (2007). Optic pathways gliomas: a review. Neurosurg Focus.

[2] Aihara Y, Chiba K, Eguchi S, Amano K, Kawamata T (2018). Pediatric optic pathway/hypothalamic glioma. Neurol Med Chir (Tokyo).

[3] Kornreich L, Blaser S, Schwarz M, Shuper A, Vishne TH, Cohen IJ (2001). Optic pathway glioma: correlation of imaging findings with the
presence of neurofibromatosis. AJNR Am J Neuroradiol.

[4] Kleihues P, Burger PC, Scheithauer BW (1993). The new WHO classification of brain tumours. Brain Pathol.

[5] Czyzyl E, Jóźwiak S, Roszkowski M, Schwartz RA (2003). Optic pathway gliomas in children with and without
neurofibromatosis 1. J Child Neurol.

[6] Albers AC, Gutmann DH (2009). Gliomas in patients with neurofibromatosis type 1. Expert Rev Neurother.

[7] Avery RA, Fisher MJ, Liu GT (2001). Optic pathway gliomas. J Neuroophthalmol.

[8] Calixto NC (2016). Gliomas de vias ópticas e estudo volumétrico por
ressonância magnética: a quimioterapia importa?.

[9] Wisoff JH (1992). Management of optic pathway tumors of childhood. Neurosurg Clin N Am.

[10] Ellis BD, Tasman W, Jaeger EA (2006). Duane’s Ophthalmology.

